# Associations of diet quality and food consumption with serum biomarkers for lipid and amino acid metabolism in Finnish children: the PANIC study

**DOI:** 10.1007/s00394-023-03293-8

**Published:** 2023-12-21

**Authors:** Suvi E. Laamanen, Aino-Maija Eloranta, Eero A. Haapala, Taisa Sallinen, Ursula Schwab, Timo A. Lakka

**Affiliations:** 1 Institute of Public Health and Clinical Nutrition, School of Medicine, University of Eastern Finland, Kuopio, Finland; 2 Institute of Biomedicine, School of Medicine, University of Eastern Finland, Kuopio, Finland; 3 Department of Medicine, Endocrinology and Clinical Nutrition, Kuopio University Hospital, Kuopio, Finland; 4 Faculty of Sport and Health Sciences, University of Jyväskylä, Jyväskylä, Finland; 5 Department of Clinical Physiology and Nuclear Medicine, Kuopio University Hospital, Kuopio, Finland; 6 Kuopio Research Institute of Exercise Medicine, Kuopio, Finland

**Keywords:** Diet, Fatty acids, Amino acids, Apolipoproteins, Lipoprotein particle size, Children

## Abstract

**Purpose:**

To investigate the associations of overall diet quality and dietary factors with serum biomarkers for lipid and amino acid metabolism in a general population of children.

**Methods:**

We studied 194 girls and 209 boys aged 6–8 years participating in the Physical Activity and Nutrition in Children study. Food consumption was assessed by 4-day food records and diet quality was quantified by the Finnish Children Healthy Eating Index (FCHEI). Fasting serum fatty acids, amino acids, apolipoproteins, as well as lipoprotein particle sizes were analyzed with high-throughput nuclear magnetic resonance spectroscopy. Data were analyzed using linear regression adjusted for age, sex, and body fat percentage.

**Results:**

FCHEI was directly associated with the ratio of polyunsaturated (PUFA) to saturated fatty acids (SFA) (PUFA/SFA), the ratio of PUFA to monounsaturated fatty acids (MUFA) (PUFA/MUFA), the ratio of PUFA to total fatty acids (FA) (PUFA%), the ratio of omega-3-fatty acids to total FA (omega-3 FA%), and inversely associated with the ratio of MUFA to total FA (MUFA%), alanine, glycine, histidine and very-low density lipoprotein (VLDL) particle size. Consumption of vegetable oils and vegetable-oil-based margarine (≥ 60% fat) was directly associated with PUFA/SFA, PUFA/MUFA, PUFA%, the ratio of omega-6 FA to total FA (omega-6 FA%), and inversely associated with SFA, MUFA, SFA to total FA (SFA%), MUFA%, alanine and VLDL particle size. Consumption of high-fiber grain products directly associated with PUFA/SFA, PUFA/MUFA, omega-3 FA%, omega-6 FA%, PUFA% and inversely associated with SFA and SFA%. Fish consumption directly related to omega-3 FA and omega-3 FA%. Consumption of sugary products was directly associated with histidine and VLDL particle size. Vegetable, fruit, and berry consumption had direct associations with VLDL particle size and the ratio of apolipoprotein B to apolipoprotein A1. Consumption of low fat (< 1%) milk was directly associated with phenylalanine. A higher consumption of high-fat (≥ 1%) milk was associated with lower serum MUFA/SFA and higher SFA%. Sausage consumption was directly related to SFA% and histidine. Red meat consumption was inversely associated with glycine.

**Conclusions:**

Better diet quality, higher in intake of dietary sources of unsaturated fat and fiber, and lower in sugary product intake were associated with more favorable levels of serum biomarkers for lipid and amino acid metabolism independent of adiposity.

**Trial Registration:**

ClinicalTrials.gov: NCT01803776, registered March 3, 2013.

## Introduction

Poor diet quality is associated with metabolic disturbances, including increased body fat content, dyslipidemia, higher blood pressure and metabolic syndrome in adults [1] and children [2, 3]. Moreover, a diet abundant with unhealthy choices, such as a high consumption of sugar-sweetened beverages and a low consumption of fruits and vegetables, has been linked to an increased risk of cardiometabolic diseases in adults [4]. Metabolomics, the study of small molecules in biofluids, provides an opportunity to explore the possible mediating factors in the complex pathophysiological pathways between diet and health [5].

In cross-sectional and cohort studies among adults, increased serum, or plasma concentrations of saturated fatty acids (SFA), aromatic amino acids (AAA), branched-chain amino acids (BCAA) and apolipoprotein (apo) B, a small low-density lipoprotein (LDL) particle size, and a large very-low-density (VLDL) particle size have been linked to metabolic disturbances and cardiometabolic diseases [6–10]. Furthermore, increased serum or plasma concentrations of SFA, AAA, and BCAA as well as a small LDL particle size have been associated with insulin resistance, an atherogenic lipid profile, and cardiometabolic risk score in cross-sectional studies among children [11–13].

Better diet quality has been linked to a more cardioprotective metabolic profile characterized as higher concentrations of serum or plasma polyunsaturated fatty acids (PUFA), lower serum concentrations of BCAA, AAA [10] and apoB [14], a smaller VLDL particle size, and a larger LDL particle size in adults [15]. However, the evidence in children on the associations between diet and metabolites, which might be the mediating factors between diet and health, is scarce. Since diet quality is relatively low among children globally [16, 17], and dietary habits track from childhood to adulthood [18], research focusing on children’s nutrition and health is extremely important in the prevention of multiple diseases and in improving overall public health. Therefore, we wanted to perform a study examining multiple dietary factors and metabolites in a large population sample of children.

We examined the associations of overall diet quality, assessed by the Finnish Children Healthy Eating Index (FCHEI), and single dietary factors with serum concentrations of fatty acids, amino acids, apoB and apoA1, as well as the sizes of VLDL, LDL, and high-density lipoprotein (HDL) particles, measured by a high-throughput nuclear magnetic resonance (NMR) spectroscopy metabolomics analysis, in a population sample of 6–8-year-old Finnish children.

## Materials and methods

### Study population

These cross-sectional analyses are based on the baseline data of the Physical Activity and Nutrition in Children (PANIC) study, which is an 8-year physical activity and diet intervention study with an ongoing follow-up in a population sample of children from the city of Kuopio, Finland. The study has been described in more detail elsewhere [19]. In short, 736 children were invited to participate in the baseline examinations of the study between 2007 and 2009. Of all invited, 512 (70%) attended. The PANIC study protocol was approved by the Research Ethics Committee of the Hospital District of Northern Savo in 2006 (Statement 69/2006). The parents or caregivers gave their written informed consent, and the children provided their assent to participation. The present study sample consisted of 403 children (194 girls, 209 boys) with complete data on variables used in the analyses, except linoleic acid for which data from 382 children were available.

### Assessment of diet

Food consumption and energy intake were assessed by food records filled out by the parents or caregivers on four predefined consecutive days including either two weekdays and two weekend days (99.5% of participants) or three weekdays and one weekend day (0.5% of participants), as described earlier [19]. At the first study visit, a clinical nutritionist instructed the parents to record all food and drinks consumed by their child at home and outside home. At the second study visit, the clinical nutritionist reviewed and completed, if necessary, the food records together with the parents. Food records were analyzed using the Micro Nutrica^®^ dietary analysis software, version 2.5 (The Social Insurance Institution of Finland), that uses Finnish and international data on the nutrient compositions of foods [20] and that was regularly updated by a clinical nutritionist.

We computed FCHEI [21] to assess overall diet quality. FCHEI consists of five categories: (1) vegetables, fruit, and berries, (2) vegetable oils and vegetable-oil-based margarine (≥ 60% fat), (3) low-fat (< 1%) milk, (4) fish, and (5) foods with high sugar content. The scoring of the index has been described elsewhere [22]. Briefly, the consumption of these foods was divided by energy intake and categorized to deciles. Deciles were scored, a higher decile getting a higher score apart from sugary products that were inversely scored. The sum of scores from the five categories was calculated, with a minimum of 0 indicating lower diet quality and a maximum of 50 indicating better diet quality.

### Biochemical analyses

Venous blood samples were collected from the children after a 12-h fast. Blood samples were centrifuged and stored at a temperature of − 75 °C until analyses. Serum concentrations of total fatty acids (FA), SFA, monounsaturated fatty acids (MUFA), PUFA, omega-3-fatty acids, omega-6-fatty acids, docosahexaenoic acid (DHA), linoleic acid (LA), alanine, glutamine, glycine, histidine, isoleucine, leucine, valine, total BCAA (including isoleucine, leucine and valine), phenylalanine, tyrosine, apoB and apoA1 as well as the sizes of VLDL, LDL, and HDL particles were measured using high-throughput NMR spectroscopy metabolomics analysis (Nightingale Health Ltd, Kuopio Finland) as described in detail earlier [23]. Finally, the degree of serum fatty acid unsaturation (i.e., the presence and amount of double bonds in the carbon chains of the fatty acids), the ratios of MUFA to SFA (MUFA/SFA), PUFA to SFA (PUFA/SFA), PUFA to MUFA (PUFA/MUFA), SFA to total FA (expressed as a percentage, SFA%), MUFA to total FA (expressed as a percentage, MUFA%,), PUFA to total FA (expressed as a percentage, PUFA%), omega-3-FA to total FA (expressed as a percentage, omega-3-FA%), omega-6-FA to total FA (expressed as a percentage, omega-6-FA%), DHA to total FA (expressed as a percentage, DHA%), LA to total FA (expressed as a percentage, LA%), and the ratio of apoB to apoA1 (apoB/apoA1) were assessed.

### Other assessments

Body height and weight were measured, and body mass index (BMI) and body mass index standard deviation score (BMI-SDS) were calculated [19]. Body fat percentage was assessed by a dual energy X-ray absorptiometry (DXA) method with a Lunar^®^ DXA device (Lunar Prodigy Advance; GE Medical Systems, Madison, WI, USA).

### Statistical analyses

The sample size of this study is based on the power calculations for our study on the intervention effects on fasting insulin and homeostatic model assessment of insulin resistance in children [24]. Briefly, we determined the number of children required to detect at least 0.30 standard deviation difference between the intervention group (60% of children) and the control group (40% of children) with a power of 80% and a two-sided *p* value for the difference between the groups of 0.05 allowing for a 20% loss to follow-up or missing data. These power calculations provided a required sample size of at least 275 children in the intervention group and 183 children in the control group. Data were analyzed using the SPSS Statistics software, version 27.0 (IBM Corporation, IBM SPSS Statistics for Windows, Armonk, NY, USA). Differences and associations with *p* values of < 0.05 were considered statistically significant. All continuous variables were checked for normality by observing histograms and using the Kolmogorov–Smirnov test. If not normally distributed, variables were transformed with logarithmic or square root transformation. Not all variables were normally distributed after these transformations, and non-parametric tests were used. To compare basic characteristics, dietary factors and metabolites between boys and girls, we used the Student’s *t* test for normally distributed variables or the Mann–Whitney’s *U* test for variables with skewed distributions.

Linear regression analyses adjusted for age and sex were used to examine the associations of FCHEI and food consumption with serum biomarkers of lipid and amino acid metabolism. For statistically significant associations, we further adjusted the data for body fat percentage that may confound or mediate the observed associations [25, 26]. We used the non-transformed dietary variables as the residuals of the linear regression analyses were normally distributed. Of the outcome variables, only serum MUFA was used as logarithmically transformed. Benjamini–Hochberg False Discovery Rate (FDR) using the FDR value of 0.2 was used to adjust results for multiple comparison [27].

## Results

### Basic characteristics and serum metabolites in children

Boys were taller, had lower body fat percentage and higher energy intake, consumed more red meat and sausages and had higher serum PUFA/SFA, PUFA/MUFA, PUFA%, omega-6 FA% and LA% than girls (Table 1). Girls had higher serum total FA, SFA, MUFA, MUFA/SFA, MUFA%, glutamine, apoB and apoB/apoA1 and lower serum apoA1 than boys (Table 2).

**Table 1:** Characteristics of children, the physical activity and nutrition PANIC study

	All (*n* 403)			Girls (*n* 194)			Boys (*n* 209)			*p* value
	Mean	SD	Median* (IQR)	Mean	SD	Median* (IQR)	Mean	SD	Median* (IQR)	
Age (years)	7.63	0.38		7.62	0.39		7.64	0.38		0.670
Height (cm)	129	5.5		128	5.7		130	5.3		**0.007**
Weight (cm)	26.9	4.8		26.7	5.2		27.0	4.5		0.501
BMI (kg/m^2^)	16.1	2.0		16.2	2.2		16.0	1.9		0.490
BMI SDS	− 0.2	1.1		− 0.2	1.1		− 0.2	1.1		0.473
Body fat percentage	19.6	8.1		22.5	7.7		16.9	7.5		** < 0.001**
Energy intake (kcal/d)	1641	308		1550	284		1724	306		** < 0.001**
Finnish Children Healthy Eating Index score	23.2	6.9		23.6	6.5		22.9	7.2		0.267
Vegetables, fruit, and berries (g/d)	211	114		216	110		206	118		0.103
Vegetable oils and vegetable-oil-based margarine (≥ 60% fat)	11.2	9.1		10.7	8.6		11.6	9.6		0.636
Low-fat (< 1%) milk (g/d)	379		372 (510)	347		350 (492)	408		426 (544)	0.075
High-fat (≥ 1%) milk (g/d)	189		94.3 (234)	182		92.4 (227)	196		99.6 (250)	0.546
High-fat (≥ 1%) sour milk products (g/d)	85.7		68.8 (100)	78.8		70.4 (88.4)	92.1		62.5 (112.5)	0.432
Fish (g/d)	15.4		7.30 (24.6)	12.7		4.9 (22.0)	18.0		8.23 (28.1)	0.133
Red meat (g/d)	56.2	30.5		51.3	26.3		60.8			**0.007**
Sausages (g/d)	22.2		15.6 (28.3)	18.1		12.1 (26.9)	26.0		18.0 (26.2)	**0.001**
High-fiber (≥ 5%) grain products (g/d)	63.3	39.4		60.9	36.4		65.6	42.0		0.343
Low-fiber (< 5%) grain products (g/d)	114	53.5		108	45.4		119	59.7		0.102
Sugary products (g/d)	190	138		176	129		203	144		0.081

Normally distributed variables were analyzed with student’s *t* test and skewed variables with Mann–Whitney *U* test

*p* values are for the difference between girls and boys, bold indicates * p* values of < 0.05 were considered statistically significant

*For skewed variables, the medians and interquartile range (IQR) (reported in parenthesis) is presented instead of SD (standard deviation)

**Table 2:** Concentrations of serum metabolites in children at baseline

	All (n 403)		Girls (n 194)		Boys (209)		p value
	Mean	SD	Mean	SD	Mean	SD	
Fatty acids							
Total FA (mmol/l)	11.181	1.115	11.298	1.138	11.073	1.084	**0.043**
SFA (mmol/l)	3.749	0.412	3.793	0.406	3.708	0.414	**0.039**
MUFA (mmol/l)	2.617	0.377	2.667	0.381	2.570	0.367	**0.007**
PUFA (mmol/l)	4.816	0.446	4.838	0.468	4.795	0.425	0.335
Omega-3 FA (mmol/l)	0.425	0.108	0.426	0.111	0.423	0.105	0.790
Omega-6 FA (mmol/l)	4.391	0.373	4.412	0.389	4.371	0.357	0.280
DHA (mmol/l)	0.357	0.169	0.369	0.187	0.346	0.150	0.181
LA (mmol/l)	3.500	0.479	3.502	0.515	3.500	0.445	0.959
Degree of unsaturation	1.248	0.078	1.251	0.080	1.246	0.080	0.488
MUFA/SFA	0.697	0.042	0.702	0.044	0.693	0.040	**0.021**
PUFA/SFA	1.290	0.097	1.278	0.095	1.301	0.099	**0.010**
PUFA/MUFA	1.859	0.200	1.830	0.196	1.886	0.201	**0.002**
SFA/total FA (%)	39.578	33.515	33.569	1.100	33.465	1.146	0.353
MUFA/total FA (%)	23.335	1.532	23.545	1.519	23.140	1.523	**0.008**
PUFA/total FA (%)	43.150	2.206	42.886	2.127	43.395	2.254	**0.021**
Omega-3 FA/total FA (%)	3.784	0.812	3.752	0.792	3.814	0.831	0.446
Omega-6 FA/total FA (%)	39.366	1.929	39.135	1.871	39.581	1.962	**0.020**
DHA/total FA (%)	3.203	1.500	1.634	0.117	1.366	0.094	0.353
LA/total FA (%)	31.304	3.005	30.944	3.426	31.635	2.521	**0.026**
Amino acids							
Alanine (µmol/l)	288.494	56.204	292.673	53.244	284.616	58.680	0.149
Glutamine (µmol/l)	678.423	60.829	685.800	63.821	671.575	58.236	**0.019**
Glycine (µmol/l)	269.692	44.975	267.258	40.244	270.024	48.986	0.538
Histidine (µmol/l))	91.661	7.688	92.171	7.893	91.187	7.482	0.200
Total concentration of BCAA (µmol/l)	387.547	55.240	390.733	54.836	384.590	55.579	0.265
Isoleucine (µmol/l)	51.384	10.429	52.132	11.104	50.690	9.737	0.166
Leucine (µmol/l)	110.683	17.226	111.387	17.012	110.029	17.438	0.430
Valine (µmol/l)	225.480	30.622	227.213	30.160	223.870	31.030	0.274
Phenylalanine (µmol/l)	64.831	6.950	65.172	6.845	64.154	7.049	0.343
Tyrosine (µmol/l)	62.533	9.806	63.397	9.333	61.731	10.182	0.088
Average diameter of lipoprotein particles							
VLDL (nm)	37.415	0.895	37.477	0.893	37.358	0.896	0.183
LDL (nm)	23.676	0.058	23.678	0.058	23.675	0.058	0.572
HDL (nm)	9.737	0.143	9.728	0.154	9.746	0.131	0.217
Apolipoproteins							
Apolipoprotein B (g/l)	0.781	0.151	0.810	0.158	0.758	0.142	**0.002**
Apolipoprotein A1 (g/l)	1.48	0.188	1.45	0.192	1.50	0.183	**0.017**
Apolipoprotein B/apolipoprotein A1	0.535	0.116	0.562	0.126	0.511	0.100	** < 0.001**

*p* values are for the difference between girls and boys, bold indicates *p* values of < 0.05 were considered statistically significant

*BCAA* branched-chain amino acids, *DHA* docosahexaenoic acid, *FA* fatty acids, *HDL* high-density lipoprotein, *LA* linoleic acid, *LDL* low-density lipoprotein, *MUFA* monounsaturated fatty acids, *PUFA* polyunsaturated fatty acids, *SFA* saturated fatty acids, *VLDL* very low-density lipoprotein

### Associations of FCHEI and single dietary factors with serum fatty acids

A higher FCHEI score was associated with higher serum PUFA/SFA, PUFA/MUFA, PUFA% and omega-3 FA% as well as lower MUFA% after adjustment for age and sex (Table 4). A higher consumption of low-fat (< 1%) milk was associated with a higher serum DHA (Table 3). A higher consumption of vegetable oils and vegetable-oil-based margarine (≥ 60% fat) was associated with higher PUFA/SFA, PUFA/MUFA, PUFA%, omega-6 FA%, and lower serum SFA, MUFA, and lower ratios of SFA and MUFA to total FA. In addition, a higher consumption of high-fiber grain products was associated with higher serum PUFA/SFA, PUFA/MUFA, PUFA%, omega-3 FA%, omega-6 FA%, and lower serum SFA, MUFA, and SFA%. A higher consumption of low-fiber grain products was associated with lower serum omega-3 fatty acids and a lower degree of serum fatty acid unsaturation. A higher consumption of high-fat (≥ 1%) milk was associated with lower serum MUFA/SFA and higher SFA%. A higher fish consumption was associated with higher serum omega-3 fatty acids and the ratio of omega-3 FA%. A higher consumption of sugary products was associated with higher SFA%. Last, higher consumption of sausages was associated with lower PUFA/SFA and higher SFA%. The associations of FCHEI with higher serum, PUFA/SFA, PUFA/MUFA, PUFA%, omega-3 FA%, and lower MUFA%, the associations of vegetable oils and vegetable-oil-based margarine (≥ 60% fat) and high-fiber grain products with higher serum PUFA/SFA, PUFA/MUFA, PUFA%, omega-6 FA%, and with lower serum SFA and SFA% remained statistically significant after further adjustment for body fat percentage (Tables 3 and 4). In addition, the association of vegetable oils and vegetable-oil-based margarine (≥ 60% fat) with lower MUFA%, the association of high-fiber grain products with higher serum omega-3 FA%, the association of high-fat (≥ 1%) milk with MUFA/SFA and SFA%, the association of fish consumption with serum omega-3 fatty acids and omega-3 FA%, and the association of sausage consumption with SFA% remained statistically significant after further adjustment for body fat percentage. The associations of FCHEI with PUFA/SFA, PUFA/MUFA, MUFA%, PUFA%, omega-3 FA%, the associations of vegetable oils and vegetable-oil-based margarine (≥ 60% fat) with SFA, MUFA, PUFA/SFA, PUFA/MUFA, SFA%, MUFA%, PUFA%, omega-6 FA%, and the associations of high-fiber grain products with SFA, MUFA, PUFA/SFA, PUFA/MUFA, SFA%, PUFA%, omega-3 FA% and with omega 6-FA% remained statistically significant after FDR correction.

**Table 3:** Associations of overall diet quality and food consumption with serum fatty acids in children

	Total FA (mmol/l)	SFA (mmol/l)	MUFA (mmol/l)	PUFA (mmol/l)	Omega-3 FA (mmol/l)	Omega-6 FA (mmol/l)	DHA (mmol/l)	LA (mmol/l)	Degree of unsaturation
Finnish Children Healthy Eating Index score	− 0.057 (0.256)	− 0.080 (0.108)	− 0.094 (0.058)	0.005 (0.924)	0.089 (0.075)	− 0.020 (0.690)	− 0.005 (0.921)	0.006 (0.905)	0.025 (0.618)
Vegetables, fruit, and berries (g/d)	0.074 (0.137)	0.068 (0.173)	0.057 (0.250)	0.065 (0.196)	0.079 (0.114)	0.055 (0.276)	− 0.004 (0.932)	0.096 (0.063)	0.037 (0.464)
Vegetable oils and vegetable-oil-based margarine (≥ 60% fat)	− 0.070 (0.160)	− 0.114^a,b^ **(0.022)**	− 0.104^b^ **(0.036)**	0.023 (0.648)	− 0.062 (0.220)	0.045 (0.368)	− 0.075 (0.136)	0.024 (0.639)	− 0.006 (0.900)
Low-fat (< 1%) milk (g/d)	0.024 (0.630)	0.029 (0.558)	0.037 (0.465)	− 0.002 (0.974)	0.070 (0.166)	− 0.022 (0.661)	0.101 (**0.043**)	− 0.008 (0.884)	0.052 (0.302)
High-fat (≥ 1%) milk (g/d)	0.008 (0.870)	0.046 (0.351)	− 0.009 (0.862)	− 0.007 (0.888)	− 0.039 (0.438)	0.003 (0.956)	− 0.076 (0.126)	− 0.042 (0.410)	− 0.039 (0.437)
High-fat (≥ 1%) sour milk products (g/d)	− 0.018 (0.716)	− 0.005 (0.914)	0.021 (0.669)	− 0.062 (0.216)	− 0.055 (0.271)	− 0.058 (0.245)	0.020 (0.688)	− 0.054 (0.297)	− 0.083 (0.098)
Fish (g/d)	− 0.012 (0.819)	− 0.022 (0.654)	− 0.036 (0.471)	0.014 (0.777)	0.107^a^ **(0.033)**	− 0.014 (0.783)	− 0.028 (0.582)	0.020 (0.706)	0.018 (0.718)
Red meat (g/d)	− 0.003 (0.945)	0.004 (0.931)	− 0.025 (0.623)	0.005 (0.925)	0.004 (0.942)	0.005 (0.927)	0.011 (0.824)	− 0.023 (0.652)	0.003 (0.957)
Sausages (g/d)	0.046 (0.358)	0.080 (0.114)	0.055 (0.277)	− 0.004 (0.938)	− 0.045 (0.377)	0.008 (0.871)	0.022 (0.671)	0.004 (0.933)	− 0.003 (0.961)
High-fiber (≥ 5%) grain products (g/d)	− 0.078 (0.119)	− 0.109^a,b^ (**0.029**)	− 0.101^a,b^ (**0.042**)	− 0.010 (0.838)	0.062 (0.214)	− 0.030 (0.546)	− 0.004 (0.933)	− 0.022 (0.671)	0.041 (0.412)
Low-fiber (< 5%) grain products (g/d)	− 0.077 (0.122)	− 0.089 (0.076)	− 0.069 (0.169)	− 0.060 (0.231)	− 0.103 (**0.040**)	− 0.042 (0.400)	− 0.067 (0.184)	− 0.010 (0.854)	− 0.106 (**0.035**)
Sugary products (g/d)	0.050 (0.320)	0.059 (0.245)	0.080 (0.110)	0.005 (0.922)	− 0.069 (0.173)	0.026 (0.609)	0.023 (0.646)	0.054 (0.299)	− 0.009 (0.855)

Data are standardized regression coefficients from linear regression analyses adjusted for age and sex

*p* values are reported in parentheses, significant values are bolded

*DHA* docosahexaenoic acid, *FA* fatty acids, *LA* linoleic acid, *MUFA* monounsaturated fatty acids, *PUFA* polyunsaturated fatty acids, *SFA* saturated fatty acids

^a^Associations remained statistically significant after additional adjustment for body fat percentage

^b^Associations remained statistically significant after Benjamini–Hochberg correction for multiple testing

**Table 4:** Associations of overall diet quality and food consumption with serum fatty acid ratios in children

	MUFA/SFA	PUFA/ SFA	PUFA/ MUFA	SFA/total FA (%)	MUFA/total FA (%)	PUFA/total FA (%)	Omega-3 FA/ total FA (%)	Omega-6 FA/ total FA (%)	DHA/total FA (%)	LA/total FA (%)
Finnish Children Healthy Eating Index score	− 0.064 (0.204)	0.119^a,b^ **(0.017)**	0.119^a,b^ **(0.016)**	− 0.092 (0.067)	− 0.107^a,b^ **(0.031)**	0.121^a,b^ **(0.015)**	0.134^a,b^ **(0.008)**	0.082 (0.099)	0.041 (0.425)	0.060 (0.244)
Vegetables, fruit, and berries (g/d)	− 0.027 (0.591)	− 0.002 (0.963)	− 0.015 (0.770)	− 0.008 (0.877)	0.023 (0.650)	− 0.012 (0.813)	0.058 (0.247)	− 0.038 (0.477)	− 0.020 (0.695)	0.069 (0.180)
Vegetable oils and vegetable-oil-based margarine (≥ 60% fat)	− 0.050 (0.361)	0.169^a,b^ **(0.001)**	(0.148)^a,b^ **(0.003)**	− 0.152^a,b^ **(0.002)**	− 0.125^a,b^ **(0.012)**	0.164^a,b^ **(0.001)**	− 0.040 (0.424)	0.205^a,b^ **(< 0.001)**	− 0.054 (0.277)	0.085 (0.096)
Low-fat (< 1%) milk (g/d)	0.006 (0.900)	− 0.036 (0.473)	− 0.045 (0.373)	0.022 (0.658)	0.048 (0.333)	− 0.045 (0.369)	0.159 (0.071)	− 0.081 (0.104)	0.097 (0.053)	− 0.035 (0.502)
High-fat (≥ 1%) milk (g/d)	− 0.118^a^ **(0.017)**	− 0.088 (0.076)	− 0.001 (0.988)	0.131^a^ **(0.008)**	− 0.044 (0.375)	− 0.036 (0.466)	− 0.053 (0.293)	− 0.019 (0.697)	− 0.079 (0.112)	− 0.073 (0.155)
High-fat (≥ 1%) sour milk products (g/d)	0.060 (0.227)	− 0.064 (0.198)	− 0.076 (0.127)	0.037 (0.466)	0.076 (0.126)	0.072 (0.151)	− 0.059 (0.241)	− 0.057 (0.253)	0.026 (0.602)	− 0.051 (0.317)
Fish (g/d)	− 0.031 (0.531)	0.050 (0.319)	0.055 (0.272)	− 0.034 (0.497)	− 0.048 (0.340)	0.051 (0.313)	0.132^a^ **(0.009)**	0.002 (0.961)	− 0.023 (0.653)	0.021 (0.686)
Red meat (g/d)	− 0.056 (0.266)	0.003 (0.958)	0.031 (0.533)	0.022 (0.671)	− 0.042 (0.400)	0.018 (0.715)	− 0.006 (0.903)	0.024 (0.639)	0.017 (0.739)	− 0.033 (0.524)
Sausages (g/d)	− 0.016 (0.756)	− 0.106 **(0.035)**	− 0.061 (0.223)	0.117^a^ **(0.021)**	0.045 (0.376)	− 0.091 (0.072)	− 0.061 (0.232)	− 0.078 (0.121)	0.006 (0.905)	− 0.020 (0.694)
High-fiber (≥ 5%) grain products (g/d)	− 0.029 (0.556)	0.146^a,b^ **(0.003)**	0.122^a,b^ **(0.014)**	− 0.130^a,b^ **(0.009)**	− 0.099 (0.056)	0.132^a,b^ **(0.008)**	0.110^a,b^ **(0.028)**	0.105^a,b^ **(0.035)**	0.013 (0.789)	0.046 (0.374)
Low-fiber (< 5%) grain products (g/d)	0.006 (0.900)	0.055 (0.273)	0.042 (0.398)	− 0.060 (0.230)	− 0.024 (0.631)	0.047 (0.343)	− 0.083 (0.098)	0.089 (0.074)	− 0.055 (0.275)	0.084 (0.100)
Sugary products (g/d)	0.074 (0.145)	− 0.074 (0.141)	− 0.092 (0.066)	0.041 (0.415)	0.093 (0.065)	− 0.086 (0.090)	− 0.107 **(0.035)**	− 0.053 (0.295)	0.020 (0.690)	0.013 (0.797)

Data are standardized regression coefficients from linear regression analyses adjusted for age and sex

*p* values are reported in parentheses, significant values are bolded

*DHA* docosahexaenoic acid, *FA* fatty acids, *LA* linoleic acid, *MUFA* monounsaturated fatty acids, *PUFA* polyunsaturated fatty acids, *SFA* saturated fatty acids

^a^Associations remained statistically significant after additional adjustment for body fat percentage

^b^Associations remained statistically significant after Benjamini–Hochberg correction for multiple testing

### Associations of FCHEI and single dietary factors with serum amino acids

FCHEI was inversely associated with serum alanine, glycine, and histidine after adjustment for age and sex (Table 5). The consumption of vegetable oils and vegetable-oil-based margarine (≥ 60% fat) was associated with lower serum alanine. A higher consumption of low-fat (< 1%) milk was associated with higher serum phenylalanine and lower glutamine. A higher consumption of sugary products and sausages was associated with higher serum histidine. A higher consumption of red meat was associated with lower serum glycine. All these associations, except that between low-fat (< 1%) milk and serum glutamine, remained statistically significant after additional adjustment for body fat percentage. The associations of FCHEI with alanine, glycine, and histidine, the association of vegetable oils and vegetable-oil-based margarine (≥ 60% fat) with lower serum alanine, the association of low-fat (< 1%) milk with phenylalanine, and the association of sugary products with histidine remained statistically significant even after FDR correction.

**Table 5:** Associations of overall diet quality and food consumption with serum amino acids in children

	Alanine (µmol/l)	Glutamine (µmol/l)	Glycine (µmol/l)	Histidine (µmol/l)	Total BCAA (µmol/l)	Isoleucine (µmol/l)	Leucine (µmol/l)	Valine (µmol/l)	Phenylalanine (µmol/l)	Tyrosine (µmol/l)
Finnish Children Healthy Eating Index score	− 0.120^a,b^ **(0.017)**	− 0.094 (0.059)	− 0.100^a,b^ **(0.046)**	− 0.153^a,b^ **(0.002)**	0.021 (0.672)	0.010 (0.847)	0.024 (0.631)	0.022 (0.668)	0.002 (0.972)	0.019 (0.703)
Vegetables, fruit, and berries (g/d)	− 0.006 (0.903)	− 0.022 (0.661)	− 0.091 (0.068)	− 0.039 (0.434)	− 0.019 (0.699)	− 0.036 (0.472)	− 0.005 (0.917)	− 0.020 (0.694)	− 0.050 (0.311)	0.001 (0.979)
Vegetable oils and vegetable-oil-based margarine (≥ 60% fat)	− 0.103^a,b^ **(0.040)**	0.023 (0.644)	0.023 (0.642)	− 0.020 (0.696)	0.025 (0.614)	0.030 (0.547)	0.043 (0.390)	0.011 (0.825)	− 0.026 (0.605)	− 0.075 (0.136)
Low-fat (< 1%) milk (g/d)	− 0.095 (0.058)	− 0.106 **(0.034)**	− 0.095 (0.058)	− 0.037 (0.459)	0.077 (0.127)	0.021 (0.678)	0.079 (0.118)	0.087 (0.084)	0.155^a,b^ **(0.002)**	0.070 (0.165)
High-fat (≥ 1%) milk (g/d)	0.074 (0.140)	− 0.024 (0.628)	− 0.074 (0.137)	− 0.051 (0.310)	0.078 (0.117)	0.053 (0.285)	0.055 (0.272)	0.092 (0.065)	0.031 (0.530)	0.011 (0.825)
High-fat (≥ 1%) sour milk products (g/d)	− 0.053 (0.291)	− 0.088 (0.077)	− 0.048 (0.342)	− 0.027 (0.596)	0.018 (0.726)	0.005 (0.918)	0.043 (0.391)	0.006 (0.908)	0.040 (0.418)	0.016 (0.753)
Fish (g/d)	− 0.020 (0.692)	− 0.040 (0.423)	0.009 (0.853)	− 0.081 (0.108)	− 0.034 (0.496)	− 0.032 (0.526)	− 0.055 (0.273)	0.020 (0.693)	0.000 (0.995)	− 0.001 (0.998)
Red meat (g/d)	0.052 (0.306)	− 0.019 (0.710)	− 0.127^a^ **(0.011)**	0.030 (0.548)	0.015 (0.762)	− 0.038 (0.447)	− 0.003 (0.954)	0.042 (0.402)	− 0.005 (0.922)	0.051 (0.308)
Sausages (g/d)	0.043 (0.392)	0.034 (0.502)	0.032 (0.522)	0.127^a^ **(0.012)**	− 0.029 (0.568)	− 0.019 (0.702)	− 0.037 (0.463)	− 0.025 (0.626)	0.016 (0.757)	0.018 (0.720)
High-fiber (≥ 5%) grain products (g/d)	− 0.062 (0.212)	0.043 (0.383)	− 0.015 (0.758)	0.019 (0.698)	− 0.037 (0.459)	− 0.027 (0.588)	− 0.038 (0.445)	− 0.036 (0.471)	− 0.055 (0.265)	− 0.062 (0.212)
Low-fiber (< 5%) grain products (g/d)	− 0.081 (0.105)	0.029 (0.558)	− 0.034 (0.501)	− 0.063 (0.207)	0.024 (0.627)	0.029 (0.563)	0.040 (0.431)	0.012 (0.813)	− 0.015 (0.762)	− 0.041 (0.415)
Sugary products (g/d)	0.086 (0.091)	0.076 (0.131)	0.073 (0.147)	0.171^a,b^ **(0.001)**	− 0.033 (0.515)	− 0.051 (0.319)	− 0.032 (0.526)	− 0.024 (0.633)	0.067 (0.185)	− 0.004 (0.939)

Data are standard regression coefficients from linear regression analyses adjusted for age and sex

*p* values are reported in parentheses, significant values are bolded

*BCAA* branched chained amino acids

^a^Associations remained statistically significant after additional adjustment for body fat percentage

^b^Associations remained statistically significant after Benjamini–Hochberg correction for multiple testing

### Associations of FCHEI and single dietary factors with lipoprotein particle size

A higher FCHEI was associated with a smaller VLDL particle size after adjustment for age and sex (Table 6). A higher consumption of sugary products and a higher consumption of vegetables, fruit, and berries were associated with a larger VLDL particle size whereas a higher consumption of vegetable oils and vegetable-oil-based margarine (≥ 60% fat) was associated with a smaller VLDL particle size. Additional adjustment for body fat percentage did not affect these associations. All the associations except for the association of a higher consumption of vegetables, fruit, and berries with a larger VLDL particle size remained after FDR correction.

**Table 6:** Associations of diet quality and food consumption with lipoprotein particle size in children

	VLDL diameter (nm)	LDL diameter (nm)	HDL diameter (nm)
Finnish Healthy Eating Index score	− 0.119^a,b^ **(0.017)**	0.024 (0.633)	0.028 (0.583)
Vegetables, fruit, and berries (g/d)	0.126^a^ **(0.012)**	0.004 (0.943)	− 0.095 (0.056)
Vegetable oils and vegetable-oil-based margarine (≥ 60% fat)	− 0.134^a,b^ **(0.007)**	0.068 (0.174)	0.063 (0.209)
Low-fat (< 1%) milk (g/d)	− 0.007 (0.884)	− 0.022 (0.659)	− 0.031 (0.537)
High-fat (≥ 1%) milk (g/d)	− 0.020 (0.683)	0.030 (0.551)	0.043 (0.391)
High-fat (≥ 1%) sour milk products (g/d)	0.003 (0.945)	− 0.041 (0.417)	− 0.008 (0.878)
Fish (g/d)	− 0.034 (0.498)	− 0.047 (0.348)	− 0.019 (0.703)
Red meat (g/d)	0.025 (0.616)	− 0.014 (0.781)	− 0.054 (0.285)
Sausages (g/d)	0.094 (0.062)	− 0.007 (0.888)	0.014 (0.790)
High-fiber (≥ 5%) grain products (g/d)	− 0.046 (0.361)	− 0.032 (0.529)	− 0.010 (0.835)
Low-fiber (< 5%) grain products (g/d)	0.006 (0.903)	− 0.060 (0.229)	− 0.001 (0.981)
Sugary products (g/d)	0.138^a,b^ **(0.006)**	− 0.035 (0.488)	− 0.088 (0.083)

Data are standardized regression coefficients from linear regression analyses adjusted for age and sex

*p* values are reported in parentheses, significant values are bolded

*HDL* high-density lipoprotein, *LDL* low-density lipoprotein, *VLDL* very low-density lipoprotein

^a^Associations remained statistically significant after additional adjustment for body fat percentage

^b^Associations remained statistically significant after Benjamini–Hochberg correction for multiple testing

### Associations of FCHEI and single dietary factors with apolipoproteins

FCHEI was not associated with serum apolipoproteins after adjustment for age and sex (Table 7). However, a higher consumption of vegetables, fruit, and berries was associated with higher serum apoB/apoA1. This association remained statistically significant after further adjustment for body fat percentage but not after FDR correction.

**Table 7:** Associations of overall diet quality and food consumption with apolipoproteins in children

	Apolipoprotein B (g/l)	Apolipoprotein A1 (g/l)	Apolipoprotein B/apolipoprotein A1
Finnish Children Healthy Eating Index score	− 0.014 (0.774)	− 0.057 (0.255)	0.015 (0.756)
Vegetables, fruits, and berries (g/d)	0.085 (0.084)	− 0.072 (0.148)	0.107^a^ **(0.029)**
Vegetable oils and vegetable-oil-based margarine (≥ 60% fat)	− 0.038 (0.445)	0.052 (0.300)	− 0.071 (0.148)
Low-fat (< 1%) milk (g/d)	− 0.010 (0.847)	− 0.056 (0.261)	0.031 (0.534)
High-fat (≥ 1%) milk (g/d)	0.021 (0.678)	0.069 (0.167)	− 0.031 (0.529)
High-fat (≥ 1%) sour milk products (g/d)	− 0.037 (0.460)	− 0.028 (0.573)	− 0.017 (0.723)
Fish (g/d)	0.028 (0.570)	− 0.075 (0.136)	0.074 (0.133)
Red meat (g/d)	− 0.007 (0.892)	− 0.003 (0.947)	− 0.001 (0.986)
Sausages (g/d)	0.001 (0.992)	0.096 (0.057)	− 0.041 (0.403)
High-fiber (≥ 5%) grain products (g/d)	− 0.070 (0.155)	− 0.064 (0.201)	− 0.027 (0.579)
Low-fiber (< 5%) grain products (g/d)	− 0.085 (0.088)	− 0.025 (0.618)	− 0.056 (0.252)
Sugary products (g/d)	0.037 (0.460)	− 0.034 (0.497)	0.051 (0.303)

Data are standardized regression coefficients from linear regression analyses adjusted for age and sex

*p* values are reported in parentheses, significant values are bolded

^a^Associations remained statistically significant after additional adjustment for body fat percentage

^b^Associations remained statistically significant after Benjamini–Hochberg correction for multiple testing

## Discussion

We found several associations of overall diet quality and single dietary factors with serum biomarkers of lipid and amino acid metabolism, particularly fatty acids, in a population sample of primary school-aged children. For instance, better diet quality was associated with higher ratios of PUFA and omega-3 FA, lower serum alanine, glycine, and histidine and a smaller VLDL particle size. Higher consumption of vegetable oils and vegetable-oil-based margarine (≥ 60% fat) and high-fiber grain products were associated with higher serum PUFAs, omega-6 FA, and lower serum SFA and MUFA. In addition, consumption of high-fiber grain products was associated with higher serum omega-3 FA%. A higher fish consumption was associated with higher serum omega-3 fatty acids, whereas a higher consumption of sugary products, high-fat dairy and meat products was associated mainly with fatty acids, serum amino acids, such as higher SFA%, lower PUFA/SFA, higher serum phenylalanine and histidine, and lower glycine. Also, a higher consumption of vegetable oils and vegetable-oil-based margarine (≥ 60% fat) was associated with a smaller VLDL particle size and a higher consumption of sugary products was associated with a larger VLDL particle size. Six of the observed associations, mainly the association of dietary factors with fatty acids such as a higher consumption of low-fiber grain products with lower serum omega-3 fatty acids and a lower degree of serum fatty acid unsaturation, were partly explained by body fat percentage, suggesting that adiposity mediates these associations. Several associations remained after the correction for multiple testing. However, concerning serum fatty acids, mainly the associations of FCHEI, vegetable oils, and vegetable-oil-based margarine (≥ 60% fat) and high-fiber grain products with serum fatty acid ratios remained statistically significant after the FDR correction.

### Fatty acids

Better diet quality was mostly associated with ratios of fatty acids, such as higher PUFA/SFA, PUFA/MUFA, MUFA%, PUFA%, omega-3 FA% but not with concentrations of any separate serum fatty acids. A previous study in Brazilian children did not find associations of diet quality and serum fatty acids [28]. However, many studies have found associations particularly between better diet quality and higher circulating *n*-3-fatty acids in children [29, 30]. Our results indicate that better diet quality may enhance the ratios of fatty acids in the serum to a more favorable direction, that meaning higher PUFA concentrations in relation to MUFA, SFA or total FA. Our findings concerning the associations of a higher consumption of vegetable oils and vegetable-oil-based margarine (≥ 60% fat) with lower serum levels of SFA, and a higher fish consumption with higher serum omega-3 fatty acids are consistent with the findings of previous studies in children [31, 32]. Furthermore, the results of this study suggest that a higher consumption of vegetable oils and vegetable-oil-based margarine (≥ 60% fat) may influence the ratios of serum fatty acids to a more favorable direction, as in the case of overall diet quality. We also observed direct associations of the consumption of high-fiber grain products with serum PUFA/SFA, PUFA/MUFA, PUFA%, omega-3 FA%, omega-6 FA%, and inverse associations with serum SFA, MUFA, and SFA%. We have previously reported that high-fiber grain product consumption is directly associated with the dietary intake of PUFAs but not MUFAs or SFAs in children [33]. Thus, children who consume high-fiber grain products may have a higher PUFA intake and a lower MUFA and SFA intake which is expected to improve serum fatty acid profile. Higher blood PUFAs have been viewed as cardioprotective in adults [6] and children [34]. Our results suggest that vegetable oils and vegetable-oil-based margarine (≥ 60% fat) and fish, foods that are rich in PUFAs might be cardioprotective by improving fatty acid metabolism since childhood. It has been reported that dietary counselling from childhood to adulthood, focusing on the quality of dietary fat, improves cardiovascular health [35]. Some of the observed associations of dietary factors with serum fatty acids were explained by body fat percentage, suggesting that adiposity may mediate these associations.

### Amino acids

Studies on the relationships between dietary factors and serum or plasma amino acids in children are scarce. We observed that better overall diet quality was associated with lower serum alanine, glycine, and histidine. An intervention study showed that plasma alanine was lower in participants following a healthy Nordic diet compared with participants following a typical Danish diet in adults [36], consistently with our findings. One explanation for these findings could be that better diet quality enhances insulin sensitivity [37] and thereby increases muscle protein synthesis [38] and decreases serum alanine. Importantly, higher circulating alanine has been directly associated with cardiovascular events in adults [7]. The results of our study suggest that better diet quality could improve cardiometabolic health by influencing amino acid metabolism since childhood. However, alanine is a non-essential amino acid that can be endogenously synthetized [39]. Hence, there might be factors affecting alanine concentrations that we were not able to account for and that would explain the observed associations.

A Korean intervention study found that serum glycine decreased after following a healthy diet with less red and processed meats compared to controls following a Western diet [40]. We found inverse associations of overall diet quality and red meat consumption with serum glycine, the latter being inconsistent with the results of the Korean intervention study. Glycine is a conditionally essential amino acid and can be synthetized, like alanine, endogenously from other sources [41] and thus it is possible that serum glycine does not directly reflect dietary intake.

The observed association of better diet quality and lower serum histidine may be partly explained by the association between a higher consumption of sugary products and higher serum histidine since higher consumption of sugary products can lower the FCHEI score. Histidine is an essential amino acid derived from animal-based products, such as meat and dairy [42]. Typical sources of sugar in our study population include dairy products, such as sweetened sour milk products and ice cream [33]. Thus, it is possible that a high consumption of these products increases serum histidine in children. However, not all milk products, such as sour milk products, were associated with serum histidine. Histidine was also directly associated with sausage consumption, which is logical considering that meat is a predominant source of histidine [42]. However, the consumption of red meat was not related to serum histidine.

Phenylalanine is an aromatic amino acid whose serum concentrations have been directly associated with cardiovascular outcomes in adults [7] and with insulin resistance in children [12]. We observed a direct association between the consumption of low-fat (< 1%) milk and serum phenylalanine. Consistent results have been reported in a study examining the effects of dairy- and meat-based complementary diets among infants [43]. The consumption of milk with higher fat content was not associated with phenylalanine in our data. Finnish children have, however, been reported to consume notably more low-fat milk than milk with higher fat content [33]. Thus, it is possible that an excessive consumption of milk leads to higher serum phenylalanine although the association is logical considering that milk and dairy products are a source of phenylalanine in diets [42]. Also, a slightly higher protein content of low-fat milk may partly explain this association.

Surprisingly, our results do not indicate that there is an association between diet and BCAA concentration, which is contrary to the results of cross-sectional studies among adults [10, 44] and an intervention study among children [45]. However, in one of the cross-sectional studies [10], the association between diet and BCAA levels was not detected when analyses were repeated in a younger population (ages between 3 and 18 years). In addition, our findings are in line with the results of a dietary counselling intervention aiming to maintain a healthier diet, which had no effect on BCAA levels in children and adolescents [46]. It is possible that adults consume bigger portions and thus larger amounts of the dietary sources of BCAA’s compared to youth or children, which is then reflected in the BCAA levels in the bloodstream. This is supported by the fact that in the intervention study among children where diet was reflected in the BCAA levels [45], the children consumed notably high portions of dairy or meat. In the present study, the consumption of dietary sources of BCAA’s might have been subtle, thus not having an impact on the BCAA levels. It has also been observed that amino acid concentrations in the plasma and skeletal muscle change with age [47]. Therefore, differences in skeletal muscle and amino acid metabolism between different age groups might also explain the contrary results.

### Lipoprotein particle size

Better overall diet quality was associated with a smaller VLDL particle size, which is in line with findings among adults [15]. We have previously found that a higher FCHEI score is associated with lower triglyceride levels in Finnish children [48]. The concentration of triglycerides, especially in the liver, might influence the formation of different VLDL subfractions; for example, the larger and less dense VLDL particles contain more triglycerides [49]. It is thus possible that triglyceride levels might explain the association between better overall quality and a smaller VLDL particle size in the present study. We also found an association between a higher consumption of sugary products and a larger VLDL particle size that could be explained by a higher fructose intake related to a high sugar product consumption. We have previously observed that sugary products are the main source of sucrose in Finnish children [33]. Since fructose is a structural unit of sucrose, it is possible that the intake of fructose is higher in children consuming higher amounts of sugary products. Fructose intake has been directly associated with liver fat content in adults [50]. VLDL particles are mostly produced in and secreted from the liver [51] and elevated fatty acid content of the liver has been observed to induce the formation of larger VLDL particles [52]. Therefore, a higher intake of fructose might lead to higher secretion of larger VLDL particles from the liver through increased liver adiposity. We also found an association between a higher consumption of vegetables, fruit, and berries and a larger VLDL size. It is possible that fructose intake from this food group increases VLDL particle size. However, there might be other lifestyle factors that explain the observed association that we could not account for. The inverse association between the consumption of vegetable oils and vegetable-oil-based margarine (≥ 60% fat), rich in PUFAs and low in SFAs, and VLDL particle size in the present study might also be explained by the accumulation of liver fat since the intake of SFAs has been directly linked to liver fat content in adults and children [53, 54]. We did not observe associations of dietary factors with LDL particle size, inconsistent with previous intervention studies in children [55, 56]. In accordance with the results of some previous studies [56], we observed no associations of dietary factors with HDL particle size.

### Apolipoproteins

Lifestyle interventions have been shown to decrease circulating apoB and increase circulating apoA1 in children with obesity and hypercholesterolemia [57, 58]. We found no associations of overall diet quality or single dietary factors with serum apolipoproteins, except the direct association of vegetables, fruit, and berries consumption with apoB/apoA1, in a general population of children. Apolipoproteins are structural components of lipoprotein particles [51], mainly LDL cholesterol particles [59]. We did not find an association between overall diet quality and serum LDL cholesterol in our previous study in children [48]. Together with the results of previous studies [57, 58], our observations suggest that overall diet quality does not influence serum apolipoproteins in metabolically healthy children, but it may be seen in children with hypercholesterolemia and with elevated circulating LDL cholesterol levels. The direct association between the consumption of vegetables, fruit, and berries and apoB/apoA1 in our study might be related to fructose intake from this food group, since it has been shown that a short-term fructose intake restriction reduces serum apoB in children [60]. However, other lifestyle factors that we could not account for mediate this association.

## Study strengths and limitations

We examined a general population sample of prepubertal children who have not yet been exposed to possible confounding factors for the associations of dietary factors with serum biomarkers for lipid and amino acid metabolism, such as alcohol consumption, smoking, diseases, and medications. Moreover, food consumption was assessed comprehensively by 4-day dietary records, reviewed by clinical nutritionists. Overall diet quality was assessed using FCHEI, which has been validated in Finnish children [21] and represents accurately the dietary challenges in our study population. Also, we used high-throughput NMR spectroscopy analysis to assess the serum biomarkers of lipid and amino acid metabolism. This method is well-suited to identify blood metabolites in large study populations [5]. This study also has limitations. First, the sample size of this study was not originally based on the current research question but is based on the power calculations for our previous study on the intervention effects on fasting insulin and homeostatic model assessment of insulin resistance in children [24]. However, we estimated that 395 participants were required to detect an association with a small effect size (*f*^2^ ≥ 0.02) in a multivariate linear regression analysis at the power of 80. Second, the assessment of diet using any measure administrated by the parents or caregivers of children, including dietary records, is prone to inaccurate reporting. Also, due to the complexity of eating behavior and human metabolism, we could not consider all possible confounding factors in the statistical analyses. Thus, the results must be interpreted carefully, simultaneously reviewing other studies on the topic. We could not conclude all possible mechanisms explaining the observed associations due to the lack of research on this topic in children. Last, we cannot draw conclusions about the causality between diet and serum metabolites due to the cross-sectional nature of this study.

## Conclusion

We observed many associations of overall diet quality and single dietary factors with serum biomarkers of lipid and amino acid metabolism in children, and these associations were mainly independent of adiposity. Better overall diet quality, a higher consumption of dietary sources of unsaturated fatty acids and high-fiber grain products, and a lower consumption of sugary products were associated with higher serum PUFAs, lower serum MUFAs and SFAs, and smaller serum VLDL particles. The results indicate that good diet quality is important in improving lipid and amino acid metabolism from an early age. Further evidence from long-term diet intervention studies is warranted to confirm whether diet modification has beneficial effects on lipid and amino acid metabolism, and whether these have meaningful effects on cardiometabolic health since childhood.

## Data Availability

The PANIC study is ongoing, and therefore the data are not fully anonymized and, thus, not openly available. Part of the data can be shared by request.
